# Panel Based MALDI-TOF Tumour Profiling Is a Sensitive Method for Detecting Mutations in Clinical Non Small Cell Lung Cancer Tumour

**DOI:** 10.1371/journal.pone.0100566

**Published:** 2014-06-23

**Authors:** James L. Sherwood, Susanne Müller, Maria C. M. Orr, Marianne J. Ratcliffe, Jill Walker

**Affiliations:** 1 Personalised Healthcare & Biomarkers, AstraZeneca, Macclesfield, United Kingdom; 2 Sequenom GmbH, Hamburg, Germany; The Chinese University of Hong Kong, Hong Kong

## Abstract

**Background:**

Analysis of tumour samples for mutations is becoming increasingly important in driving personalised therapy in cancer. As more targeted therapies are developed, options to survey mutations in multiple genes in a single tumour sample will become ever more attractive and are expected to become the mainstay of molecular diagnosis in non-small cell lung cancer (NSCLC) in the future.

**Materials and Methods:**

238 non-small cell lung cancer (NSCLC) tumour samples were analysed using a custom panel of 82 mutation assays across 14 oncogenes including *KRAS* and *EGFR* using Sequenom iPlex Matrix Assisted Laser Desorption/Ionisation Time of Flight Mass Spectrometry (MALDI-TOF). We compared the data generated for *KRAS* mutations to those detected by Amplification Refractory Mutation System (ARMS) based DxS TheraScreen K-RAS Mutation Kit.

**Results:**

The ARMS detected mutations in 46/238 tumour samples. For samples with mutations detected by both approaches, 99.1% overall agreement was observed. The MALDI-TOF method detected an additional 6 samples as *KRAS* mutation positive and also provided data on concomitant mutations including *PIK3CA* and *TP53*.

**Conclusions:**

The Sequenom MALDI-TOF method provides a sensitive panel-based approach which makes efficient use of patient diagnostic samples. This technology could provide an opportunity to deliver comprehensive screening of relevant biomarkers to the clinic earlier in disease management, without the need for repeat biopsy and allow for additional downstream analysis in NSCLC where available tissue may have been exhausted.

## Introduction

NSCLC makes up about 85% of all lung cancers [1]. Approximately 20% of Caucasians with NSCLC, rising to over 26% of those with adenocarcinomas (ADC) have activating mutations in *KRAS* [2]. Asian populations have a *KRAS* mutation incidence of 11% in ADC. Mutations in *EGFR* are present in 48% of Asian NSCLC ADC versus 19% in Caucasian ADC. *EML4-ALK* mutations are present in 6% of Caucasian NSCLC ADC and 5% of Asian ADC [2]. Molecular analysis of aberrations in *EGFR* and *ALK* is well established and used to identify patients suitable for targeted therapies such as the EGFR tyrosine kinase inhibitors (TKIs) gefitinib, erlotinib and afatinib, and *ALK* inhibitors such as crizotinib [3]. *KRAS* is an important emerging marker in NSCLC. The clinical value of establishing *KRAS* mutation status may increase if the development of MEK inhibitors in NSCLC with mutant KRAS deliver positive risk benefit outcomes for patients. MEK is known to be a downstream effector of *KRAS* signalling and has been implicated in cell proliferation and tumour growth. Selumetinib (AZD6244, ARRY-142886) is a potent and selective, non-ATP-competitive MEK1/2 inhibitor [4]. A recent phase II clinical trial (NCT00890825) compared the efficacy of selumetinib in combination with docetaxel versus docetaxel alone in pre-treated patients with *KRAS* mutation-positive locally advanced or metastatic non small cell lung cancer. Median overall survival was 9.4 months (6.8–13.6) in the selumetinib group and 5.2 months (95% CI 3.8-non-calculable) in the placebo group (hazard ratio (HR) for death was0·80, 80% CI 0·56–1·14; one-sided p = 0.21). Median progression-free survival was 5·3 months (4·6–6·4) in the selumetinib group and 2.1 months (95% CI 1.4–3.7) in the placebo group (HR for progression 0.58, 80% CI 0.42–0.79; one-sided p = 0.014) [5]. The efficacy of selumetinib in wild type *KRAS* NSCLC has not yet been established. Other MEK inhibitors in development include cobimetinib (GDC-0973, XL-518) and trametinib. The latter was recently approved for use by the FDA in *BRAF* V600E mutated melanoma. Demonstration of a clear clinical benefit in a *KRAS* mutation-positive NSCLC population leading to drug approval would drive the need to identify relevant *KRAS* mutations in NSCLC patients at diagnosis, in addition to *EGFR* and *ALK* aberrations, to inform treatment decisions.

In the NCT00890825 trial the ARMS based DxS TheraScreen K-RAS Mutation Kit was used to prospectively identify *KRAS* mutation-positive patients eligible for randomisation and treatment. ARMS methodology was selected as it provides superior sensitivity and specificity in formalin fixed paraffin embedded (FFPE) material when compared to direct sequencing [6], [7]. In the clinical trial setting this qPCR based method could be performed with a rapid turn around time on small patient numbers as the samples were received.

In another recent trial of selumetinib in cutaneous melanoma, NCT00936221 [8], samples were analysed using a combination of ARMS and sequencing methodologies to test for *BRAF* mutations in codon V600.

The Sequenom iPlex Pro MALDI-TOF technology allows multiple mutations in FFPE samples to be analysed in a single investigation using multiplex PCR reactions [9]. The technology uses small (∼80 base pairs) PCR product amplification which is optimal for amplification of fragmented DNA templates such as those extracted from FFPE tumour samples. Following amplification, a single base pair extension step is performed at the site of the mutated base of interest with a mass modified ddNTP termination mix. The advantage of this approach is the ability to resolve the four bases on the spectra. The resultant fragment, with modified base at the site of mutation, is then analysed using the Sequenom MassARRAY mass spectrometer which is designed and optimised specifically for nucleic acid detection. A clear advantage of this system is the ability to identify any mutant base at the given position meaning one assay covers all three possible base changes without the need for a separate assay for each potential mutation. For example the Gly12Cys mutation in *KRAS* is caused by a G>T transversion at position 34. Sequenom iPlex Pro will detect any mutation at this base position including the Gly12Arg and Gly12Ser mutations caused by the G>C transition and the G>A tranversion respectively. This reduces the template DNA requirement and therefore minimises tissue demand.

Here we report how the MALDI-TOF method compares to ARMS as a method to detect *KRAS* and *BRAF* mutations in clinical samples and how the use of a multiplex panel allowed us to assess the prevalence of less common mutations in our NSCLC cohort.

## Materials and Methods

### Tumour Sample Analysis

The 238 NSCLC patient samples came from the NCT00890825 study [5].

177 melanoma samples from the NCT00936221 study [8] were also collected and processed as described in Robert et al [8].

All procedures were carried out in accordance with the Helsinki Declaration (1964, amended in 1975, 1983, 1989, 1996 and 2000) of the World Medical Association and all patient samples submitted for analysis were done so with the full informed consent of the patients.

### Pathology

Patients in this NSCLC cohort provided a tumour sample dating from between May 2007 and May 2010. Samples were confirmed histologically or cytologically as stage IIIB-IV NSCLC following H&E staining by a qualified histopathologist at Labcorp Centre for Molecular Biology and Pathology (CMBP), Research Triangle Park, NC, USA.

### Nucleic Acid Extraction

Nucleic acid extraction was performed at Labcorp CMBP, Research Triangle Park, NC, USA, from 4×5 µm FFPE sections, with removal of paraffin by melting.

DNA extraction was carried out using the Qiagen QIAamp DNA Mini Kit, (QIAGEN GmbH, Hilden, Germany), according to the kit insert. Elution was into 100 µL water.

DNA was quantified using the TaqMan RNase P Detection Reagents kit and TaqMan Universal PCR Mastermix (Applied Biosystems, Carlsbad, California U.S.A) to establish amplifiable yield.

### *KRAS* Mutation Status Analysis

Tumour samples were prospectively assessed for *KRAS* mutation status using the TheraScreen K-RAS ARMS Mutation Kit (QIAGEN Manchester [formerly DxS Ltd], Manchester, UK).

Quantitative Polymerase Chain Reaction (qPCR) was performed using an ABI Prism 7900 qPCR system (Applied Biosystems).

*KRAS* mutation analysis was carried out by Labcorp CMBP, Research Triangle Park, NC, USA.

An aliquot of the remaining DNA was supplied to Sequenom GmbH, Hamburg for analysis using Sequenom iPlex chemistry and MALDI-TOF.

### Sequenom iPlex Pro Assay Design

Table 1 shows the custom panel of mutation assays which was designed for mutations in 14 oncogenes namely *BRAF, CDNK2A, CTNNB1, EGFR, ERBB2, FGFR, HRAS, KRAS, STK11, MET, NRAS, TP53, PIK3CA* and *PTEN*. Mutations were selected based on known frequency in NSCLC or melanoma, biological significance or presence of any significant “hotspots” lending themselves to targeted assays [2], [10]–[13].

**Table 1:** Mutations targeted by the MALDI-TOF Panel by gene.

Gene	Targeted Variants^a^
*BRAF*	V600E/K/L
*CDNK2A*	R58*, E61*, E69*,R80*, H83Y, D84Y, P114L, W110*
*CTNNB1*	S33F/C/Y, S37F/C/Y
*EGFR*	E746_A750DEL-2235-2249, E746_A750DEL-2236-2250, G719A/D/C/S, L858R/M, T790M
*ERBB2*	D769H, G776V, H878Y, L755S, G776V, H878Y, L755S, V777L, V842I
*FGFR*	K650E/M, R248C, S249C
*HRAS*	G12C/R/S/V/A/D, G13C/R/S, Q61L/R/P/K/E
*KRAS*	G12C/R/S/V/A/D, G13C/S/A/D, Q61E/H/L/R/P, A146A/P/V
*STK11*	Q37*, Q170*, F354L, D194Y/N,
*MET*	Y1248C, Y1253D
*NRAS*	G12G12C/R/S/V/A/D, G13C/S/A/D, Q61E/H/L/R/P
*TP53*	R175H/L, R213*, Y220C/S, G245S/C/R, R248W/G, R249S, R273C/L/H, R282W/R
*PIK3CA*	K111E/N/R, E542*/K/Q, E545A/G/D/K/Q, Q546*/H/R/P/L, H1047L/R/Q/Y
*PTEN*	R130G/R/L/P/Q, R166fs*17, R173C/P/H, R233*, R335*

a The list is not exhaustive as rarer variants can also be detected.

The mutation analysis was performed at Sequenom GmbH (Hamburg) and consisted of 10 multiplexes containing 82 assays to detect over 160 different mutations. The manufacturer's standard protocol was followed [9]. In short, 2 µL of template genomic DNA was amplified by multiplex PCR to extend wild-type (WT) and mutant DNA, followed by a shrimp-alkaline-phosphatase treatment to remove surplus nucleotides. Next, a primer extension reaction (iPLEX Pro) was performed with mass-modified terminator nucleotides, and the products were spotted on a SpectroCHIP (Sequenom). The distinct masses were determined by MALDI-TOF mass-spectrometry. Data were analyzed using MassARRAY Typer Analyser software (Sequenom) [9].

## Results

### Analysis Success Rates

DNA yield ranged from ∼10 genomic copies per µL to ∼30,000 copies per µL with a mean of 1837 copies per µL (5.9 ng/µL). Eleven samples with very low copy number (≤10 copies per µL) failed analysis using the ARMS kit. All samples were successfully analysed using the MALDI-TOF panel.

### Comparison Between MALDI-TOF Panel and ARMS Kit for *KRAS* in NSCLC

238 samples were analysed using the ARMS kit which was designed to detect the following 7 *KRAS* mutations: G12C/R/S/V/A/D and G13D. 46/238 (19%) patient samples were classified as *KRAS* mutant positive by the ARMS test. The MALDI-TOF method covers G12C/R/S/V/A/D, G13D/V/A/E and Q61L/R/P/E/K/H. 53/238 (22%) samples were classified as *KRAS* positive by MALDI-TOF, with one patient having two separate mutations, one in codon 12 and one in codon 61.

A *KRAS* A146 mutation assay was included on the MALDI-TOF panel and no mutations were detected, consistent with previous reports suggesting this particular mutation, although relevant in colorectal cancer, is not important in NSCLC [12], [14].

### Concordance of MALDI-TOF Method with ARMS Kit

Concordance between the two platforms was determined using data from samples that were evaluable by both methods (i.e. we excluded the 11 samples that failed by ARMS method). The MALDI-TOF method detected 6 (13%) more *KRAS* mutations in total, in part due to broader coverage (Table 2 & 3). For those mutations detectable by both platforms the positive percentage agreement (PPA) between the ARMS method and MALDI-TOF method was 97.8% (Table 4) and the overall percentage agreement (OPA) was 99.1%. The specificity of the Sequenom platform in samples established as wild type by ARMS was 98.3% (negative percentage agreement).

**Table 2:** Comparison of number of individual *KRAS* mutations detected by MALDI-TOF and ARMS kit.

Mutation detected	MALDI-TOF	ARMS kit
G12C	24	25
G12D	13	11
G12V	6	6
G12A	3	3
G13D	2	1
G13C	2	Not tested
Q61H	2	Not tested
Q61E	1^a^	Not tested
Total	53	46

a One sample contained two *KRAS* mutations, one in codon 12 and one in codon 61.

**Table 3:** Number of *KRAS* mutations detected vs. mutation not detected (MND) and fails by both methods.

	MALDI-TOF MS	ARMS kit.
*KRAS* +	52	46
MND	186	181
Fail	0	11

**Table 4:** Number of NSCLC samples in agreement between ARMS kit and MALDI-TOF in assays common to both kits.

		ARMS kit		
		*KRAS*+	MND	Total
MALDI-TOF MS	*KRAS*+	45	1^a^	46
	MND	1^b^	176	177
	Total	46	177	223
	Positive Percentage Agreement (PPA) = 97.8%			
	Negative Percentage Agreement (NPA) = 98.3%			
	Overall Percentage Agreement (OPA) = 99.1%			

a Figure does not include samples where they failed *KRAS* ARMS analysis.

The two discordant samples included one sample in which MALDI-TOF detected a G12D mutation with the ARMS assay result exceeding the criteria described in the kit insert for a positive result. In the second sample, MALDI-TOF generated no detectable signal whilst ARMS detected a G12C. The most likely explanation for this is that the high proportion of wild type DNA may have exceeded the analytical sensitivity of standard iPlex Pro chemistry. It has been shown by this group (data not published) that this panel was able to detect mutations robustly down to 5% (limit of our analysis) using cell line admixtures.

Therefore, the Sequenom MassARRAY platform performs at least as well in (OPA = 99.1%) assessing *KRAS* mutation status in clinically available NSCLC FFPE samples as the ARMS platform (Table 4), with enhanced analytical sensitivity (Table 3).

### Prevalence of Other Mutations in NCT00890825 NSCLC Cohort

MALDI-TOF data was generated on additional mutations including codon 12, 13 and 61 in *HRAS* and *NRAS* and codon 600 in *BRAF*. 107 of the 238 samples (45%) were positive for at least one mutation (see Table S1). 52/238 (22%) of patients had mutations in *KRAS*, consistent with expected prevalence in NSCLC [2] (Table 5). *NRAS* mutations were detected in 5 patients (2.1%) including 3 Q61 mutations, a G12 and a G13 mutation. There were 3 *BRAF* V600E mutant samples and 2 *HRAS* mutant samples in codons Q61 and G12.

**Table 5:** Mutation prevalence in NCT00890825 NSCLC patients using MALDI-TOF panel compared to COSMIC NSCLC database.

Gene	Mutation prevalence (of 238 analysed)	COSMIC Frequency for NSCLC
KRAS	21.8%	17%
TP53	9.7%	26%
EGFR	8.8%	25%
PIK3CA	4.2%	3%
CDNK2A	2.5%	14%
NRAS	2.1%	1%
BRAF	1.3%	1%
PTEN	1.3%	1%
HRAS	0.8%	<1%
STK11	0.42%	5%
MET	0%	3%
ERBB2	0%	1%
CTNNB1	0%	1%

A number of known *TP53* mutations were investigated and found to be present in 23/238 (10%) of patients. It should be noted that the MALDI-TOF assay only interrogated 10/480 (2%) of substitutions that have been reported in lung by systematic screens in the COSMIC database [13], although they were the 10 most common mutated bases reported therein (Table 5).

*EGFR* mutations were detected in 20/238 (8.8%) of our samples. This is a little lower than expected from previous reports that suggest *EGFR* mutations are present from 10% to 15% in Caucasians with NSCLC [15]. A number of factors may have contributed to the low prevalence of *EGFR* mutations in our study. Firstly 9% of our samples were squamous cell carcinoma, which have much lower incidence of *EGFR* mutations than ADC. Secondly, we cannot rule out local pre-selection of patients for our study, due to knowledge of *KRAS* positive status following local testing, *EGFR* positive subjects being diverted towards TKI therapy or increased proportion of subjects with history of smoking, which would be less likely to have *EGFR* mutations [2], [12].

### Concomitance of Mutations in NSCLC

**Figure 1:**
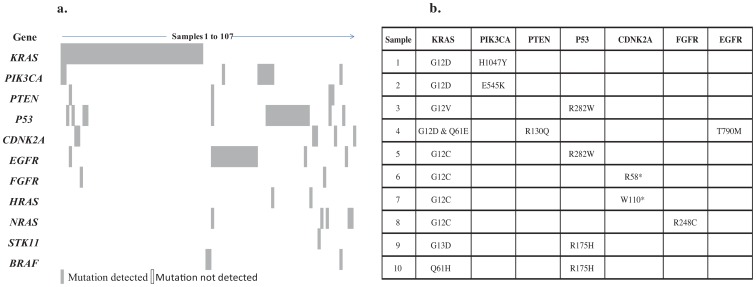
NSCLC samples 1-107 with mutations detected in each gene. B). *KRAS* mutated samples in NCT00890825 with concomitant mutations.

10/52 (19%) of patients with *KRAS* mutations had concomitant mutations, the most common of which were *TP53*, *PIK3CA* and *CDNK2A*. Figure 1a. shows the concomitance of mutations in each tumour sample that contained at least one mutation, n = 107.

Figure 1b. shows the 10 samples which were *KRAS* positive and had concomitant mutations. Four samples had *TP53* mutations which were either in the *TP53* R282 or the R175 DNA binding domain mutations. Both these mutations are in the top 6 most frequent *TP53* mutations found in cancer and are deleterious to TP53 function [16].

Two samples had a *KRAS* G12D mutation co-occurring with an activating *PIK3CA* mutation. Concomitance of these mutations has been observed previously [12]. One sample contained an R248C mutation in the *FGFR* extra-cellular domain which was coincidental with *KRAS* G12C. *FGFR3* mutations are present at around 3% in lung cancer [17] and the R248C mutation is known to drive tumour formation in xenograft models which were inhibited by multi-kinase inhibitor ponatinib.

One sample with a *KRAS* mutation showed a concomitant mutation with an *EGFR* T790M mutation. *KRAS* and *EGFR* activating mutations are generally considered to be mutually exclusive [18] nonetheless they have been observed at very low frequencies [19], [20]. The T790M mutation is known to confer resistance to *EGFR* TKI's. Unusually, this sample also contained a double *KRAS* mutation and a *PTEN* mutation. It should be noted that the concentration of DNA was extremely low in this sample and further confirmation by sequencing was not possible.

### Performance of the MALDI-TOF MS Panel in Melanoma Samples

The same MALDI-TOF MS panel was also used on a set of 177 melanoma samples from clinical trial NCT00936221 which assessed the efficacy of selumetinib in combination with dacarbazine compared with dacarbazine alone in first line patients with *BRAF* mutation positive advanced cutaneous or unknown primary melanoma [8]. 69/177 (39%) samples were *BRAF* V600E positive using MALDI-TOF. This compared very well with the results from NCT00936221, which was performed using a combination of two different ARMS based tests and Sanger sequencing, which detected BRAF V600E mutations in 69/177 samples. The vast majority of samples were investigated using the ARMS tests. Two samples were *BRAF* mutation positive by MALDI-TOF and negative by Sanger sequencing. The MALDI-TOF spectrographs indicated that these mutations were present below the nominal sensitivity of Sanger sequencing which can be as high as 20% mutant DNA in wild type background. Two samples were negative by MALDI-TOF but positive by an ARMS based test. The ARMS test had a 2% sensitivity, which is better than the 5% sensitivity we established for the MALDI-TOF panel (data not shown). Overall 21/177 (12%) of the melanoma samples failed analysis using either an ARMS test or Sanger sequencing, whereas the MALDI-TOF method was successful in all cases. *NRAS* mutations were also detected in 38/177 (22%) melanoma samples using the MALDI-TOF panel. As a direct comparison against the data generated by ARMS and by sequencing collectively the MALDI-TOF the overall percentage agreement was 97% (152/156).

## Discussion

In this study, we investigated the utility of the Sequenom MassARRAY MALDI-TOF platform in mutation detection in NSCLC and melanoma, compared with ARMS technology which has shown superior sensitivity than direct sequencing in FFPE samples. The MALDI-TOF method had a higher analysis success rate in samples with low DNA yield, most likely due to the smaller amplicon size (∼80 bp), well below the average fragment size of DNA obtainable from FFPE samples (∼200 bp). Previous studies have established the analytical sensitivity of Sequenom's Mass Spectrometry technology as 1–10% [21]. This data shows that Sequenom's MALDI-TOF technology is of sufficient sensitivity and specificity to be used to direct therapy for *KRAS* mutation positive patients, in NSCLC.

The importance of testing for mutations in the 3 codons (G12, G13 and Q61) in *KRAS* commonly mutated in NSCLC was demonstrated by the detection of 13% more *KRAS* positive patients in this clinical cohort. A phase III trial, SELECT-1, (NCT01933932) is currently underway to assess efficacy and safety of selumetinib in combination with docetaxel in *KRAS* mutation positive NSCLC patients receiving 2nd line treatment. In this trial, patients are being selected using the cobas *KRAS* Mutation Test (CE/IVD) which is designed to detect 19 mutations in codons 12, 13 and 61 [22].

Other mutations in *HRAS*, *NRAS* and *BRAF* were also detected. Since these mutations have been linked to MEK activation, patients carrying these mutations might also be candidates for treatment with a MEK inhibitor although the significance of these mutations in NSCLC is unknown [23]–[25]. For example lung cancer cell lines containing *NRAS* mutations have been shown to be sensitive to the MEK inhibitor selumetinib [26].

The data generated for *BRAF* V600E mutations in melanoma samples also showed concordance in 152/156 (97%) of samples with ARMS or Sanger sequencing demonstrating the accuracy of the MALDI-TOF MS method in *BRAF* V600E mutations. Cutaneous melanoma samples often contain high concentrations of melanin which inhibits PCR. The successful analysis of samples which failed using ARMS demonstrated the ability of the MALDI-TOF MS method to successfully yield data in melanoma samples.

A future challenge in the clinic is the probability of reduced tissue availability due to increased demand for testing. Sequential reflex testing of genetic aberrations can result in the need for further invasive biopsy procedures which could be avoided in some cases by testing for all informative markers in parallel when the patient first presents with NSCLC.

The value of the Sequenom platform was that it used less sample than other methods; 2 times less DNA was used to genotype 27 times as many loci (82 vs 3) across 14 genes, than was used for the ARMS test that looked only at a single gene. Conceivably 2×5 µm sections eluted into 50 µL would have sufficient material for the molecular characterisation of the samples in this cohort. The data in our study was generated in 1 working day indicating its tractability in the clinic in consideration of turn around requirements.

We detected mutations concomitant with *KRAS* in 10/52 (19%) of cases. Two samples showed concurrent *PIK3CA* mutations with *KRAS*. *PIK3CA* mutations in combination with *KRAS* have been shown to confer resistance to MEK inhibitors *in-vivo* [27]. The ability to detect concomitant mutations could provide additional clinical benefit and potentially offer an insight into appropriate combination approaches to therapy. The patient numbers in NCT00890825 with concomitant mutations were too small to observe any link to response.

Our data demonstrates the potential utility of the Sequenom MALDI-TOF panel in clinical NSCLC samples.

A potential limiting factor to the use of panel tests such as Sequenom in the diagnostic setting is the requirement by regulatory authorities for extensive validation of each detectable variant which is not always straight forward due to availability of samples for validation.

It should be noted that use of this technology to detect mutations in tumour suppressor genes such as *TP53* and *CDNK2A* would be an impractical and inefficient use of tissue due to the number of bases that would need to be interrogated.

Next Generation Sequencing (NGS) technology can also cover multiple genes. NGS is especially advantageous when screening genes that have disparate mutation patterns such as *TP53*, and can detect novel mutations, something less readily achieved on the MALDI-TOF platform. NGS panels generally require a higher DNA input than this system to enable generation of useable sequencing libraries, and sensitivities may be limited to around 5% depending on read depth requirements and sequence context. NGS also presents its own challenges in turn around time and costs for analysis, curation and data management.

In a diagnostic setting where few mutations are of known relevance to targeted therapies, such as in NSCLC, Sequenom MALDI-TOF has a distinct advantage over direct sequencing methods due to its proven utility in FFPE derived DNA, fast turn around time, modest data storage requirements and minimal user analysis requirement.

In conclusion, the Sequenom MALDI-TOF approach to mutation profiling is an efficient and informative use of patient samples. It shows comparable sensitivity and good concordance with a well established extremely sensitive *KRAS* ARMS test when used in NSCLC samples, with the ability to identify a wider range of mutations, using less tissue. Concomitant mutation profiles in NSCLC FFPE samples could inform the treatment strategy that clinicians use on an individual patient basis.

## Supporting Information

Table S1 All mutations detected by the customised MALDI-TOF panel, by sample.
(XLSX)
